# Functional Haplotypes and Evolutionary Insight into the Granule-Bound Starch Synthase II (*GBSSII*) Gene in Korean Rice Accessions (KRICE_CORE)

**DOI:** 10.3390/foods10102359

**Published:** 2021-10-03

**Authors:** Thant Zin Maung, Sang-Ho Chu, Yong-Jin Park

**Affiliations:** 1 Department of Plant Resources, College of Industrial Science, Kongju National University, Yesan 32439, Korea; tzmaung.yau2009@gmail.com; 2 Center of Crop Breeding on Omics and Artificial Intelligence, Kongju National University, Yesan 32439, Korea; sanghochu76@gmail.com

**Keywords:** granule-bound starch synthase 2 (*GBSSII*), haplotype, SNP, domestication, cultivated rice, wild rice

## Abstract

Granule-bound starch synthase 2 (*GBSSII*), a paralogous isoform of *GBSSI*, carries out amylose biosynthesis in rice. Unlike *GBSSI*, it mainly functions in transient organs, such as leaves. Despite many reports on the starch gene family, little is known about the genetics and genomics of *GBSSII*. Haplotype analysis was conducted to unveil genetic variations (SNPs and InDels) of *GBSSII* (*OS07G0412100*) and it was also performed to gain evolutionary insight through genetic diversity, population genetic structure, and phylogenetic analyses using the KRICE_CORE set (475 rice accessions). Thirty nonsynonymous SNPs (nsSNPs) were detected across the diverse *GBSSII* coding regions, representing 38 haplotypes, including 13 cultivated, 21 wild, and 4 mixed (a combination of cultivated and wild) varieties. The cultivated haplotypes (C_1–C_13) contained more nsSNPs across the *GBSSII* genomic region than the wild varieties. Nucleotide diversity analysis highlighted the higher diversity values of the cultivated varieties (weedy = 0.0102, landrace = 0.0093, and bred = 0.0066) than the wild group (0.0045). The cultivated varieties exhibited no reduction in diversity during domestication. Diversity reduction in the japonica and the wild groups was evidenced by the negative Tajima’s *D* values under purifying selection, suggesting the domestication signatures of *GBSSII*; however, balancing selection was indicated by positive Tajima’s *D* values in indica. Principal component analysis and population genetics analyses estimated the ambiguous evolutionary relationships among the cultivated and wild rice groups, indicating highly diverse structural features of the rice accessions within the *GBSSII* genomic region. *F_ST_* analysis differentiated most of the classified populations in a range of greater *F_ST_* values. Our findings provide evolutionary insights into *GBSSII* and, consequently, a molecular breeding program can be implemented for selecting desired traits using these diverse nonsynonymous (functional) alleles.

## 1. Introduction

Rice is a cereal grain in which the starch is mainly used as a food source for humans. Starch biosynthesis includes many enzymes, such as starch synthase, granule bound starch synthase (GBSS), starch-branching enzyme (SBE), debranching enzyme, and pullulanase, which synthesize amylose and amylopectin, the two main components of starch [1]. GBSS is the only enzyme that synthesizes amylose, which is a linear form of starch. GBSS has two similar isoforms, GBSSI which carries out amylose synthesis in the endosperm [2], and GBSSII which has the same activity but in leaves [3]. Salt-induced research has been conducted on the down-regulation of *GBSSI* and *II* genes to achieve molecular regulation of starch accumulation in rice seedling leaves [4]. 

Starch granules accumulate in plants as transient starch in the chloroplasts of source organs (such as leaves) or storage starch in amyloplasts of sink organs (such as seeds, tubers, and roots) [5]. Transient starch is synthesized during photosynthesis, whereas storage starch accumulates once photosynthesis becomes inactive and later degrades to sustain the seedlings during germination [6]. Comprehensive expression analysis revealed that GBSSII protein is mainly expressed in non-storage tissues of plants, such as leaves [7], for starch biosynthesis. *GBSSII* functions as a mediating binding protein during starch granule development in rice leaves [8]. Similar to the *GBSSI* gene, which is a grain quality (eating and cooking quality) determinant of amylose content in the seed endosperm, the *GBSSII* gene expressed in rice leaves also induces nitrogen starvation by supplying ammonia or amino acids, which, in turn, affect the photosynthetic products (hexokinase inhibitor) [7].

It was recently discovered that the location of GBSS on starch granules in Arabidopsis leaves is facilitated by the presence of the protein targeting to starch 1 (PTST1) protein, which is specifically required for amylose biosynthesis [9]. PTST1 was first identified as a plastidial protein containing an N-terminal coiled-coil domain (specialized α-helices that often mediate protein-protein interactions) and a carbohydrate-binding module 48 (CBM48) at the C-terminus [9,10]. Seung et al. [11] updated their findings with two additional plastidial proteins, PTST2 and PTST3, which are homologs of PTST1, and identified their expression in chloroplasts of Arabidopsis leaves, where they control the initiation of small starch granules.

Despite the differences in specific expression patterns and protein locations of *GBSSII* and *GBSSI* [3], the two genes encoding these proteins share high sequence similarity with almost 66% identical deduced amino acid sequences in wheat [3] and about 70% homology at the nucleotide sequence level in rice [4]. However, several different functional alleles or haplotypes [12,13,14] have been identified in the waxy gene (*GBSSI*) region and are associated with amylose content in rice and many other crops, such as maize [15], wheat [16], barley [17], and cassava [18,19], but research on the functional properties of *GBSSII* gene in crops is limited, particularly in rice. A recent study reported novel *GBSSII* haplotypes in rice through genome-wide identification and genetic variation analyses [20], but additional evolutionary analyses on this gene have not been performed. Evolutionary insights based on SNP haplotypes of many starch synthase genes (*GBSSI*, *SSSI*, *SSIIa*, *SSIIb*, *SSIIIa*, *SSIIIb*, *SSIVa*, and *SSIVb*) other than *GBSSII* have been provided by the high variability between cultivated and wild rice populations [21]. The limited findings on *GBSSII* genetics and its evolutionary history allowed us to explore the genomic region of this gene using high-throughput sequencing technology in the 475 KRICE_CORE accession set based on classified rice populations.

Next-generation sequencing (NGS) is a powerful tool to discover domestication genes in crop plants and their wild relatives [22,23]. The recent rapid development of NGS has boosted the number of genomic sequences for hundreds to thousands of rice varieties [24]. Evolutionary research on rice has been performed previously (Khush 1997) and various strategies, such as whole-genome resequencing [25] or sequencing [26,27], natural or artificial selection [12], and genotyping-by-sequencing [16], have been explored by breeders to broaden the genetic diversity of crops by examining sequence variations and either evolutionary relationships between or among populations using NGS data. Genetic variations (alleles) have been detected using various selection methods and have been increasing in number due to their distinctive patterns or signatures due to selection or other confounding effects, such as bottlenecks, expansions, or subdivided populations [28].

A breeding program to develop a new cultivar requires knowledge and understanding of the genetic diversity, genetic relationships, and population structure of the desired gene [29]. To identify the functional genetic properties of *GBSSII* gene and provide evolutionary insight, a deep sequencing analysis was performed for the whole genomes of the 475 Korean rice accessions (KRICE_CORE), and genetic variations were detected. Moreover, a series of analyses was conducted to estimate the genetic diversity, population genetic structure, and genetic relationships using classified rice populations. Notably, limited information is available about the functional properties of the *GBSSII* gene and the related evolutionary story in rice. The findings of this study will fill in missing information on the functional properties of the *GBSSII* gene and its domestication signatures, which, in turn, will be applicable to future rice variety breeding programs.

## 2. Materials and Methods

### 2.1. Plant Materials and Experimental Site

A core set of 421 cultivated rice accessions consisting of three variety types (landrace, weedy, and bred) (Data S1) was collected previously around the globe, administered by the National GeneBank of the Rural Development Administration (RDA-GenBank, Republic of Korea) using the PowerCore program [30], and selected for whole-genome resequencing [31]. An additional set of 54 wild rice accessions was obtained from the International Rice Research Institute in 2017. These two core sets were combined to form the KRICE_CORE (Korean World Rice Collection).

Field experiments were conducted with these 421 Asian-cultivated and 54 wild-rice accessions in the departmental field of the Plant Resources Department, Kongju National University (Yesan Campus) in 2016 and 2017. Among the three varieties, there were 279 temperate japonica, 26 tropical japonica, 102 indica, 9 aus, 2 aromatic, and 3 admixed varieties (Table S1). Recommended cultural practices for crop management were carried out as necessary.

### 2.2. DNA Extraction, Resequencing and Variant Calling

Plant samples (young green leaves) were collected from all tested plants approximately 15 days after transplanting. DNA was extracted using the cetyltrimethylammonium bromide (CTAB) method, and genomic DNA was stored at 4 °C until use [32]. High-quality DNA was used for whole-genome resequencing of the rice varieties with an average coverage of approximately 15× on the Illumina HiSeq 2000 Sequencing Systems Platform. These HiSeq 2000 sequencing data were deposited in the NCBI GenBank data (accession numbers: SAMEA4999071—SAMEA4999480, SAMN12714206—SAMN12714236 and SAMN16203520—SAMN16203712). The decoded sequences were saved in the FastQ file format. A program package, VCFtools (variant call format) version 0.1.15 [33], was used to remove missing values and heterozygotes from the raw data and the results were saved in the FastQ format. To compare the output sequences among the accessions, high-quality reads that remained after removing the missing values and heterozygotes were aligned to the International Rice Genome Sequencing Project (IRGSP) 1.0 rice genome sequence. The alignment of the reads was saved in binary alignment map (BAM) format. Duplicate reads aligned in multiple locations were removed using PICARD (version 1.88) software [34]. SNP and InDel calling was then performed using GATK tools (version 4.0.1.2) [35,36] to identify the SNP regions in the BAM file. The extracted mutations were saved in VCF file format and filtered using VCFtools (version 0.1.15) to remove false-positive SNPs/InDels. To identify the genetic variants, the specific variant files of the classified subgroups were viewed by using bcftools program package version 1.8 and their respective numbers of genetic variants were counted in TASSEL 5 (version 20210408) software [37].

### 2.3. Population Genetic Structure and Phylogenetic Study

To identify the number of populations in the 475 rice accessions, VCFtools version 0.1.15 was used to convert the previously called variants into PLINK outputs, and bed files were created again using the PLINK (version 1.07) analysis toolset. Two additional files (.bim and .fam format) were incorporated using Python script (structure.py) with the fastStructure [38] package tools and a range of increasing K values from 2 to 7. The admixture patterns of the defined populations (population structure) were inferred using average Q-values in the POPHELPER version 2.3.1 [39] analytical tool of RStudio (version 1.4.1106) software. Principal component analysis (PCA) was processed to explore the classified subpopulations on a dimensional scale in RStudio version 1.4.1106. A list of principal components (PC1 and PC2) referring to variants was generated in TASSEL 5 (version 20210408) [37], and relatedness among the classified subpopulations was investigated in 2D scatterplots using RStudio (version 1.4.1106). A phylogenetic analysis was conducted in MEGA X software [34] using the neighbor-joining method, and a tree was drawn in FigTree (version 1.4.4) software.

### 2.4. Nucleotide Diversity, Tajima’s D, and the Fixation Index (F_ST_ Test)

To determine the degree of polymorphism within the pairwise comparisons of the classified populations, the resulted DNA sequences based on the populations were investigated for their diversity using nucleotide diversity values (π). Using these population-based sequences with variants, Tajima’s *D* values were also calculated to measure the difference between the estimated average number of nucleotides and the observed number of segregating sites for all 475 rice accessions. The estimated measures of the fixation index (*F_ST_*-values) were investigated to observe the performance of the genetic relationship between and among the populations. Using VCFtools (version 0.1.15), variant files were selected for the *GBSSII* gene region to compare the classified representative cultivated ecotypes/subpopulations. The sliding window size used for nucleotide diversity (π) and the Tajima’s *D* calculations was 1.5 kb, and the values were compared among the classified rice groups based on their varietal types or ecotypes.

### 2.5. Haplotype Network

A haplotype network was constructed to investigate the genetic relatedness of the samples based on variants within the *GBSSII* gene region. VCFtools was used to specify the selected gene regions of the tested samples, and the reference gene sequences adapted from the Rice Annotation Project Database (RAP-DB, https://rapdb.dna.affrc.go.jp/index.html, last accessed on 20 April 2021) were aligned in Molecular Evolutionary Genetics Analysis (MEGA X) version 10.1.8 software [40]. A compiled list of aligned sequences was saved in the nexus file format and analyzed in DnaSP (version 6.0) software [41] to generate a list of haplotypes with their accession numbers. Taking the accessions with each haplotype into account, a list of the same mutated sequences was created for each trait/ecotype in Population Analysis with Reticulate Trees (PopART) software [42], and a TCS network [43] was drawn using that ecotype list.

## 3. Results

### 3.1. Identification of Genetic Variations

The *GBSSII* genomic region (*Os07g0412100*) is located between positions 12,916,883 and 12,924,202 (–strand) on chromosome 7 and is 7320 bp in length. Genetic variations among populations are important because the genetic differences are the ultimate source of useful mutations for breeding. The whole *GBSSII* genomic region of the 475 rice accessions were resequenced using VCFtools and the counted variations classified by geography (ecotypes). Five types of genetic variations were identified, including single nucleotide polymorphisms (SNPs), insertions (Ins), deletions (Dels), duplications (Dupls), and different variations (DVs) (Table 1). Among the variations, SNPs were the most frequently observed and comprised the largest number of variations in every cultivated ecotype, of which aromatic varieties had the highest number of SNPs (200) among the classified cultivated subgroups. For the specific numbers of variations, the wild rice group was subdivided into *Oryza nivara*, *Oryza rufipogon*, and others. Cultivated and wild rice have a considerable number of variations that are useful for investigating their functional responses in evolutionary analyses. Interestingly, all of the classified cultivated subgroups (ecotypes) had more SNPs, indicating differences in segregated sites, which, in turn, supported the genetic diversity values.

### 3.2. Haplotype Variations

**Figure 1:**
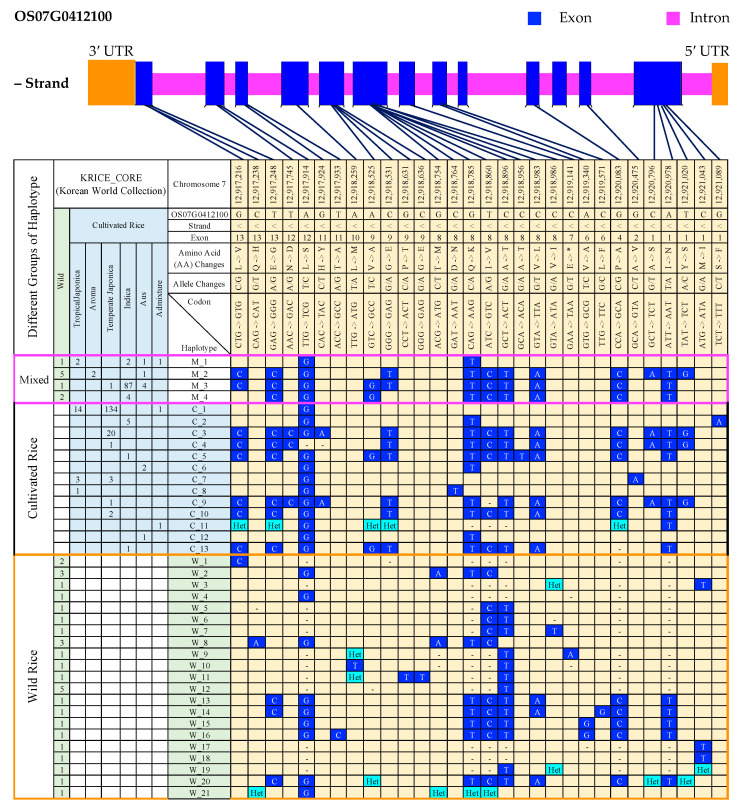
Gene structure and haplotype analysis of the *GBSSII* (*Os07g0412100*) gene in the 475 Korean rice accessions. Light-blue (cultivated) and pale-green-colored (wild) columns provide the list of haplotypes together with their respective number of rice accessions in each subpopulation. Blue cells indicate all SNP variations (minor alleles) found only at nonsynonymous mutation sites. Blank cells indicate major alleles or the same nucleotide to that of the reference. Cyan color, “Het”, refers to “heterozygote”, and the “dash (-)” indicate a position for an “unknown” nucleotide or generally refers to “N” (any nucleotide A, T, G, or C). C: cultivated rice, W: wild rice, M: a combination of cultivated and wild rice. Note: The selected number of chromosome positions and sorted haplotypes were based on the identifications where nonsynonymous substitutions (nsSNPs) or functional substitutions were observed.

To investigate the haplotype variations that were due to differences in alleles (SNPs and InDels) at their different segregating sites, a haplotype analysis was performed for all of the cultivated (421) and wild (54) rice accessions and the differences or similarities in terms of polymorphic changes (SNPs,) including InDels, were determined within the *GBSSII* gene region. Using such genetically distinct sequences of the 475 rice accessions, these analyzed sequenced accessions were grouped into haplotypes after alignment with the Nipponbarae reference (Data S2). The differences among the classified haplotypes were examined. A total of 45 haplotypes was observed representing a total of 113 variants, including 58 SNPs and 55 InDels, within the gene region of *GBSSII* in chromosome 7 (Data S3). There were 30 nonsynonymous SNPs among the 58 SNPs, and as a summary, 38 functional haplotypes were verified based on these SNPs, and mainly discussed these functional SNP substitutions (Figure 1). According to the haplotypes in which they belonged to the same rice ecotypes, it was formed into three main groups, including cultivated rice (13 haplotypes: C_1–C_13), wild rice (21 haplotypes: W_1–W_21), and mixed (cultivated and wild, 4 haplotypes: M_1–M_4).

Nineteen functional substitutions (nonsynonymous SNPs) were identified in the group of cultivated haplotypes (C_1–C_13) and were present in 162 temperate japonica, 18 tropical japonica, 6 indica, 3 aus, and 2 admixture rice accessions. Among these 19 functional SNPs, there were four C to T nucleotide substitutions (two yielded the same amino acid change and the other two produced different amino acid changes). The remaining substitutions and their frequencies were three G/A and T/C; two G/C, A/G, and C/A; G/T, A/T, and T/G indicating transitions in their respective amino acids. Among the 13 cultivated haplotypes, C_1 was the predominant haplotype (149 accessions), followed by C_3, a temperate japonica-specific haplotype represented by 20 accessions. Despite C_1 being represented by the highest number of rice accessions, there was only a single functional SNP (A/G in exon 12). By contrast, C_3 contained 14 functional SNPs, which was the highest number of alleles for any of the haplotypes.

Twenty functional SNPs were detected in the wild group (21 haplotypes: W_1–W21), representing 30 wild rice accessions. Different frequencies of functional SNPs were observed with four C/T; three C/A; two G/C; two T/C; two A/G; two A/T; and two G/T, G/A, C/G, and T/C. Thirteen functional SNPs were found in the mixed group (cultivated and wild, four haplotypes: M1–M4), representing two (G/C, T/C, A/G, C/T, C/A), G/T, A/T, and T/G for 105 cultivated and 9 wild rice accessions.

Most of the functional substitutions were in the same positions in the wild and cultivated rice groups. However, some functional SNPs were found only in the cultivated or wild groups. Such functional SNPs as T/C (exon 12), G/A (exon 11), C/T (exon 8), and G/A (exons 2 and 1) were only found in the cultivated haplotypes, with different amino acid changes. Similarly, the wild haplotypes also contained various SNPs including C/A (exon 13); T/C (exon 11); A/T (exon 10); G/T and C/T (exon 9); G/A, G/T, T/C, two C/T, and C/A (exon 8); A/G and C/G (exon 6); G/C (exon 4); and A/T and C/T (exon 1). The number of functional SNPs at different segregation sites did not differ between the cultivated and wild groups.

Despite more functional SNP numbers in the wild group, the number of representative rice accessions were very few. In case of polymorphisms by insertions or deletions (InDels), a greater number of diverse alleles was found in the wild group compared to the cultivated rice group based on the same number of 38 haplotypes (Figure S4). In this case, some of the minor alleles were responsible to minor haplotypes, especially to the wild and the positions for such alleles were excluded together with the positions indicated by 1 bp Ins/Del variations. Lastly, there were only 27 positions for InDels and interestingly, all the identified variations generated were deletions (Dels) (Figure S4). Similar to the haplotypes for SNP, major haplotypes (such as M_3 and C_3, except C_1) indicated the same deletion (Del) variations. Interestingly, most identified haplotypes generated deletion (Del) variations.

### 3.3. Population Genetic Structure and Genetic Differentiation (F_ST_ Test)

**Figure 2:**
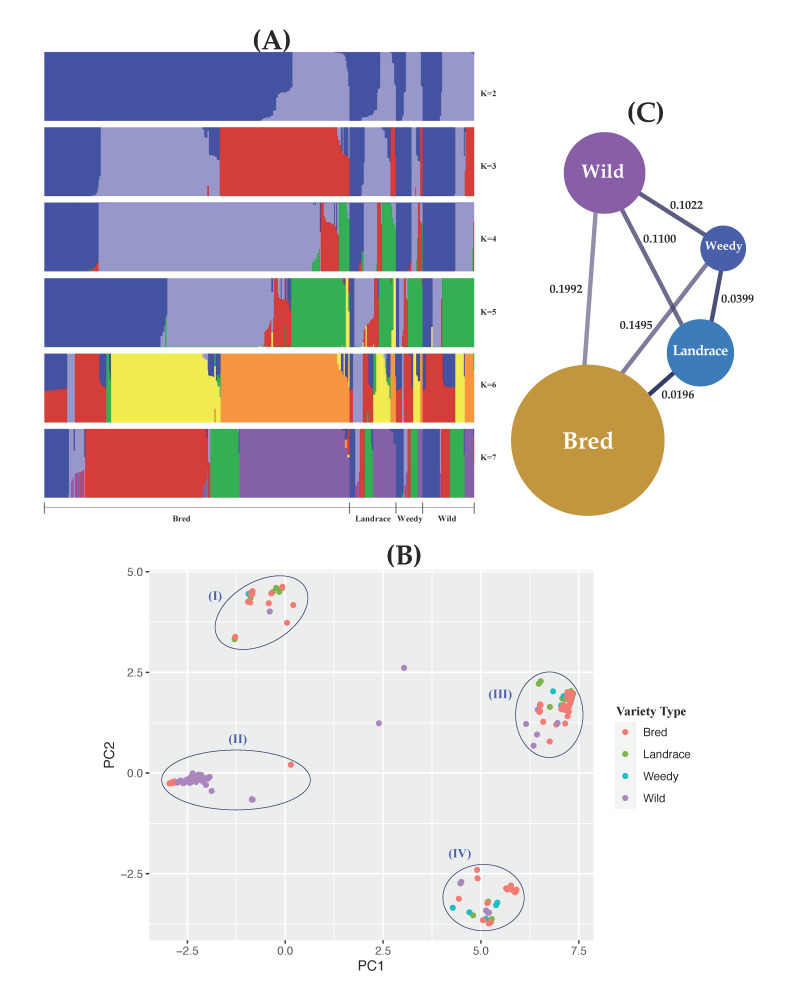
Estimate of structure and population differentiation within the gene region of *GBSSII* (*Os07g0412100*) in the Korean rice collection in terms of different varietal types (landrace, weedy, bred, and wild). (**A**) Population structure of the *GBSSII* gene in the 475 Korean rice accessions clustered by increasing K value from 2 to 7. The different colors of each K value refer to the different numbers of clustered populations. (**B**) Two-dimensional (2D) principal component analysis (PCA) of the 475 Korean rice accessions. (**C**) Pairwise estimates of genetic differentiation (*F_ST_* values) of the *GBSSII* gene among different varietal types of the 475 Korean rice accessions.

To stratify the ancestral proportion of the classified populations due to the genetic similarity or differentiation of the *GBSSII* gene, the analysis on population structure was carried out by increasing the K value from 2 to 7 (Figure 2A). At every K value (K = 2 to 7), the wild group was clearly separated from the cultivated ecotypes, but its internal subgroups were partially admixed with each other. On the variety basis, from K = 3 to 7, the cultivated subgroups were also separated from each other but at K = 3, the internal subgroups of bred and landrace were in close ancestral history indicating a high genetic similarity. At K = 3 and 4, the identified clusters of the wild enhanced a clear separation to the wild from the cultivated subgroups, but the genetic influxes within the *GBSSII* region were connected with all of the classified varieties, especially the bred variety. Meanwhile, the structural features of landrace and bred were similar at K values 4 and 5, indicating their close associations. On the basis of ecotypes, the cultivated subgroups were also separated with each other (Figure S1A), but like the wild group, their internal subgroups, by different cluster numbers, shared their ancestral proportions, indicating their close genetic association within the *GBSSII* region. Despite the clear separation at K = 3 and 4, the major cultivated ecotypes, indica and temperate japonica, had a similar population structure, and the rice accessions shared their genetic properties in the *GBSSII* region between them. Each group was subdivided into many individual subgroups at every K value, suggesting that there might be internal interbreeding events among or between the subgroups.

The genetic relatedness of classified populations was explored by performing the analysis based on the detected principal components within the *GBSSII* gene region. There were four clear separations of different clusters by *GBSSII* genetic differentiation levels (Figure 2B). As expected, the wild group revealed the most diverse *GBSSII* genetic structure, although its accessions were clearly separated as a group (group II). Among the cultivated groups, bred was shown to be an isolated group (group II) but associated with some wild accessions while its additional individuals were closely admixed with other cultivated groups, landrace and weedy in groups I, III and IV, indicating their genetic similarity within the *GBSSII* gene region. Once PCA on the cultivated rice accessions were repeated based on their ecotypes (Figure S1B), the most admixed cluster was observed in group I, exhibiting that most of the cultivated subpopulations, except admixture and aromatic, are associated with the wild group. Group III was indica specific, indicating its close relatedness to some wild rice accessions. Group IV was clustered with a higher proportion of temperate japonica, followed by indica, aromatic, and wild. Overall, four clear separate groups revealed admixed clusters in the cultivated and wild groups, suggesting a closer genetic association within the cluster.

To determine the level of genetic differentiation between the classified subpopulations, the fixation indices (*F_ST_* values) were estimated using genetic variants within *GBSSII* gene region (Figure 2C, Figure S1C and Table S2). On the basis of varieties (landrace, weedy, and bred), the differentiation levels from the wild group were signified by pairwise *F_ST_* values. The *F_ST_* values among the classified groups indicated a range from 0.0196 to 0.1992, suggesting a similar differentiation between them (Figure 2C). In case of ecotype basis, the greater genetic distances were indicated between most of the cultivated subpopulations, and the highest *F_ST_* value was detected between tropical japonica and aromatic (0.8318), followed by temperate japonica and aromatic (0.7567) (Table S2). As expected, a close association tendency by narrow genetic differentiation was observed between temperate and tropical japonica (0.0498). Genetic differentiation of the wild from the cultivated subpopulations were varied: the highest was observed in indica (0.4335) and the lowest occurred in aus (0.1343). Two major cultivated subpopulations, indica and japonica (temperate and tropical), expressed a wide range of genetic differentiation of 0.6952 for indica-temperate japonica and 0.6935 for indica-tropical japonica. Statistically, aus had a greater differentiation from both japonica groups, compared to its closer relationship with indica (Figure S1C).

### 3.4. Nucleotide Diversity Analysis

**Figure 3:**
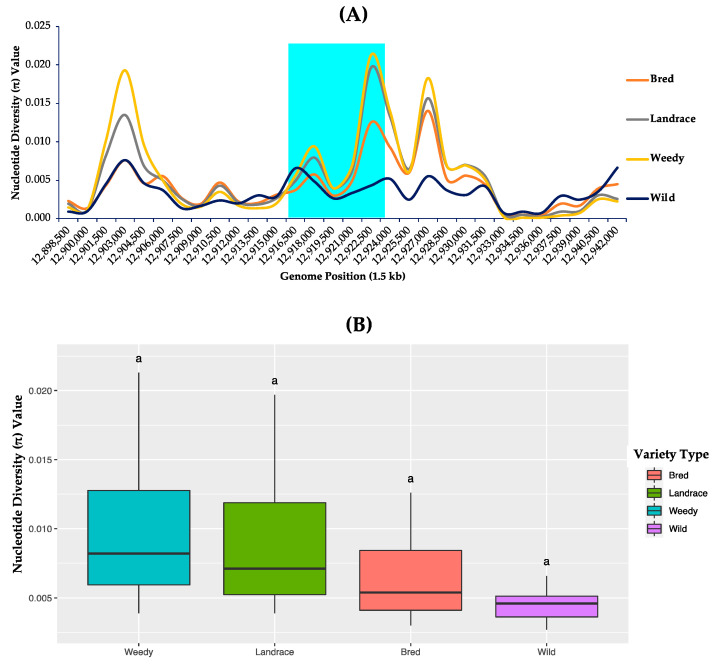
Nucleotide diversity analysis of *GBSSII* (*Os07g0412100*) in the 475 Korean rice accessions utilizing the variety type (landrace, weedy, and bred) together with the wild group. (**A**) Nucleotide diversity (π-value) representing the number of nucleotide variations within the *GBSSII* gene region at individual segregating sites in 1.5 kb sliding windows. Cyan indicates the *GBSSII* gene region, and each colored line represents a different rice varieties. (**B**) Box plots representing the different distribution patterns of *GBSSII* genetic variations based on mean nucleotide diversity values among the variety types. Lowercase letter “a” indicate a significant difference level at *p*-value < 0.05.

To measure the degree of polymorphism due to different genetic variations such as SNPs and InDels, the nucleotide diversity values of *GBSSII* were measured based on the classified populations, and the differences investigated between and among them (Figure 3 and Figure S2). Here, it was considered to include the upstream and downstream regions of *GBSSII* to estimate diversity. In the case of variety basis, landrace, weedy, bred, and wild, the resulted nucleotide diversity values ranged from the lowest of 0.0027 (wild) at the position 12,919,500 to the highest of 0.0213 (weedy) indicated at position 12,922,500 (Figure 3A). Once the nucleotide diversity values were compared by their average values (Table S3), it was noticed that the lowest diversity value (0.0045) was in the wild group, while the highest diversity value occurred in the weedy group (0.0102). Despite no significant difference in diversity values among the classified groups, a considerable reduction in diversity was found in the wild group compared to any of the cultivated rice groups.

The differences in nucleotide diversity values were investigated among the classified groups by means of ecotype differences (temperate japonica, indica, tropical japonica, aus, aromatic, and admixture), including a group for the wild rice accessions (Figure S2 and Table S4). It was observed that aus indicated the highest nucleotide diversity value (0.0332) at chromosome position of 12,922,500 and the lowest in temperate japonica (0.0003) at position 12,919,500. On the basis of comparing their average values, the same previous feature of the lowest and highest diversity values was represented by temperate japonica (0.0029) and aus (0.0141) and the wild (0.0045) was only higher than the japonica subpopulations (temperate and tropical japonica). Overall, despite the lowest value in the wild group the japonica group showed the lowest nucleotide diversity value among the ecotypes, indicating a wide range of genetic variation of *GBSSII* among the cultivated populations.

### 3.5. Tajima’s D Test

**Figure 4:**
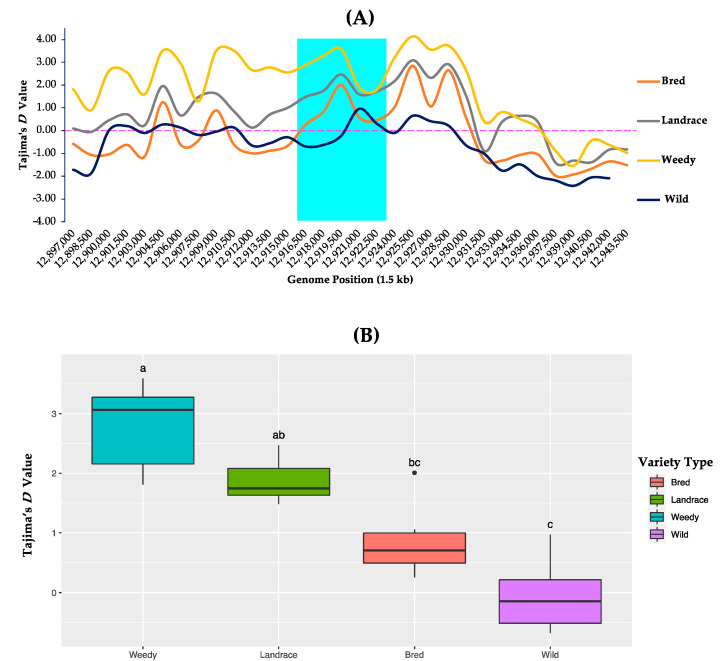
Tajima’s *D* values of *GBSSII* (*Os07g0412100*) in the 475 Korean rice accessions by means of variety types (landrace, weedy, and bred) together with the wild. (**A**) Tajima’s *D* values, representing different individual segregating sites within the *GBSSII* gene region in the 1.5 kb sliding window. Cyan indicates the *GBSSII* gene region, and each colored line represents a different rice variety type. (**B**) Box plots represent different distribution patterns of the *GBSSII* genetic variations according to the Tajima’s *D* values among the variety types. Different lowercase letters (a, ab, bc and c) indicate a significant difference level at *p*-value < 0.05.

To understand the difference between the estimated values of the nucleotide differences and the observed values of the segregating sites, the corresponded DNA sequences within the *GBSSII* gene region were analyzed to calculate Tajima’s *D* values based on the classified subpopulations of the 475 rice accessions. Tajima’s *D* values were computed using the same classified rice groups used for the nucleotide diversity analysis (Figure 4 and Figure S3). On the basis of variety type, all the cultivated varietal groups showed only positive Tajima’s *D* values within the *GBSSII* gene region while both positive and negative values were indicated for the wild group. The highest Tajima’s *D* value (3.5924) was detected at position 12,919,500 in the *GBSSII* gene region of the weedy group and the lowest (−0.6795) was found for the wild group at 12,916,500. Once the average Tajima’s *D* values were calculated, the same representative of the highest and positive Tajima’s *D* value was in the weedy rice set (2.7883), while the lowest and negative value (−0.0499) was still in the wild rice (Table S3). Interestingly, all the cultivated varietal groups had positive Tajima’s *D* values.

In case of ecotype basis, all the classified subgroups showed both positive and negative Tajima’s *D* values, except aus in which all identified positions indicated positive Tajima’s *D* values (Figure S3). From their average values within the *GBSSII* gene region, negative Tajima’s *D* values were observed in both the japonicas (−0.4769 for temperate and −0.3883 for tropical) and the lowest Tajima’s *D* value (−0.0499) was represented by the wild group. The other ecotypes exhibited positive values in their descending order, 2.0490 (aus), 0.9794 (indica), and 0.5919 (admixture) (Table S4).

### 3.6. Haplotype Network and Phylogenetic Analyses

**Figure 5:**
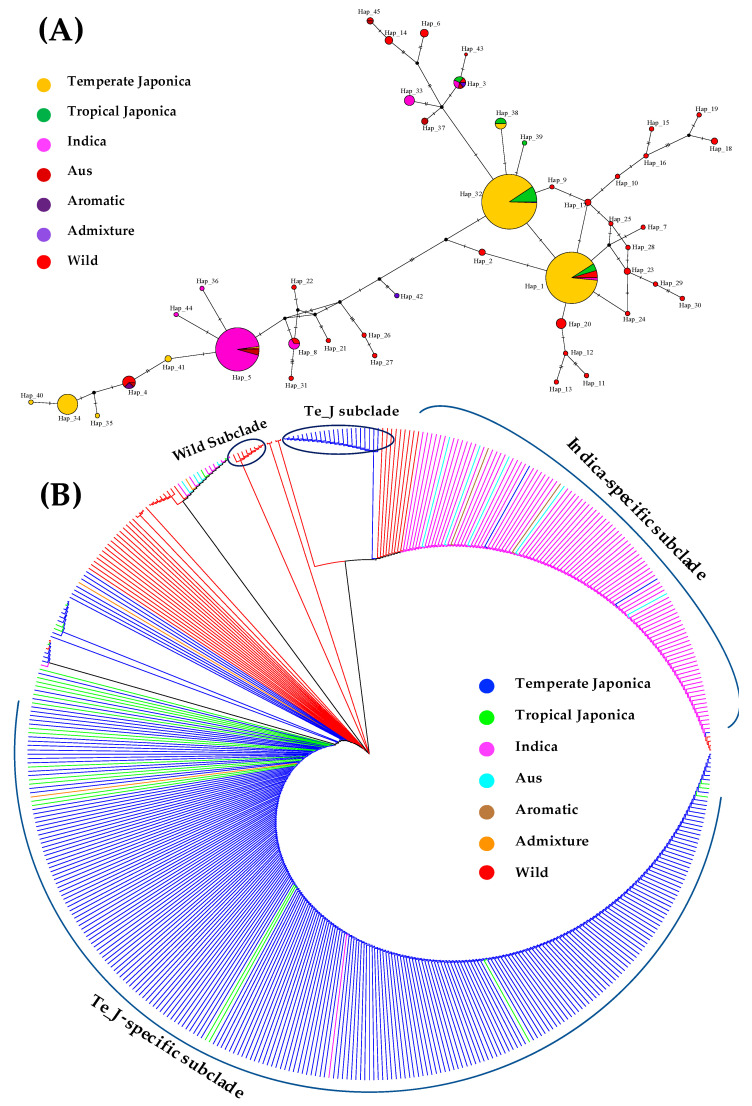
Haplotype-based analysis of the *GBSSII* (*Os07g0412100*) gene in the 475 Korean rice accessions. (**A**) Haplotype network visualizing the evolutionary relationships among the identified haplotypes. The size of each circle is proportional to the number of accessions that harbor the haplotype, and different colors refer to the different ecotypes. Black circular dots indicate median vectors and in the present analysis, there were 20 median vectors in total. (**B**) Phylogenetic tree inferring the evolutionary relationships among the tested rice accessions, indicating their ancestral history using the neighbor-joining method. Different colors refer to different ecotypes.

To visualize how the rice accessions were genetically related (different or similar) within the *GBSSII* gene region during the evolution, the informativeness of evolutionary analyses were investigated through haplotype network and phylogenetic analyses. A haplotype network was constructed to infer the level of evolutionary relationships among the different classified populations from the 475 rice accessions, using a list of generated haplotypes identified within the *GBSSII* gene region (Figure 5A). Forty-five haplotypes created a network, covering genetically different DNA sequences in the cultivated (temperate japonica, indica, tropical japonica, aus, aromatic, and admixture) and wild rice groups. As shown in Figure 5A, three major haplotypes were found. One (Hap_5) was indica specific and the other two (Hap_1 and Hap_32) were temperate japonica predominant. The temperate japonica-specific haplotypes (Hap_1 and Hap_32) were close to each other, sharing similar genetic variations of *GBSSII* with tropical japonica, indica, and the admixture including the wild rice. The indica-specific haplotype (Hap_5) was distantly associated with the temperate japonica-specific haplotypes, indicating different genetic variation of *GBSSII*, but shared its similarity with the aus, a few temperate japonica, and wild accessions. The remaining cultivated haplotypes were derivatives of these three major haplotypes at different levels of genetic variation. On the other hand, the wild haplotypes were diverse throughout the network, linking each other in closer genetic relationship under the *GBSSII* region. However, some wild haplotypes had close associations with cultivated haplotypes, particularly with temperate japonica-specific haplotypes.

To view the relationship of the tested rice accessions during the evolution of *GBSSII*, a phylogenetic analysis was conducted using different nucleotide sequences from the core set of 475 rice accessions based on the six cultivated subpopulations and one wild group (Figure 5B). It was clearly observed for two clear separations of cultivated subpopulations in the neighbor-joining tree: the temperate japonica-specific subclade shared its similar genetic variation of *GBSSII* with, of course, many tropical japonica, indica, and admixture, derived from the same common ancestor and an indica-specific subclade displayed a close association with many wild, aus, admixture and aromatic, including temperate japonica. This indica-specific subclade was rooted in the same and most recent common ancestor of another temperate japonica subclade. Both major separate subclades were derived from different wild ancestors, indicating a distant common ancestor. Despite the clear separation of the wild subclades, the relatedness of their internal rice accessions was high, and they shared similar genetic variations in *GBSSII* with cultivated rice. By contrast, not only the two major separated groups (indica and japonica) and the other cultivated rice groups (aus, aromatic, and admixture) were separate, although most of them in the same clade were under the recent common ancestors.

## 4. Discussion

Starch is composed mainly of the glucose polymers amylose and amylopectin, which are alpha-D-glucose units [44]. Most of the starch molecules are 5–35% amylose, which is synthesized by granule-bound starch synthase (GBSS) [44]. The linear chain length of amylose is elongated by GBSS. GBSS is one of the several isoforms of starch synthases (SS) of land plants with two paralogues: GBSSI, which is strictly expressed in endosperm and pollen, and another enzyme, GBSSII, which is expressed in non-storage tissues (e.g., leaves) and the pericarp [3,7,45]. GBSSI is a major determinant of cooking and eating quality based on the amylose content in the rice endosperm, whereas amylose biosynthesis is carried out in rice leaves by GBSSII [8]. Despite these differences, the two enzymes were derived from the same *Oryza species* (*O. japonica*) but belonged to different subclades of an orthologous tree based on 19 plant species, including most rice and other *GBSS*-related species (Figure S5). Comparative sequence analysis is an approach to study genome function and evolution by identifying homologous or paralogous genes to interpret their relationships [46]. *GBSSII* in our study is a paralogous of *GBSSI*, representing 70% homology in rice by deducted amino acid [4].

Genetic variations (alleles) in crop species, such as SNPs, InDels, and intron length polymorphisms (ILPs), are essential to revealing molecular mechanisms, as they function in controlling traits [47,48,49,50]. Genetic variations of the starch synthase gene family has recently been updated in rice giving new insights into their correlation with improved amylose content and eating quality [20]. Using these identified variations, genetic markers have been developed for various analyses, including genetic diversity assessment, trait association mapping, and fine mapping of QTLs that regulate important agronomic traits [51]. In this study, a core set of 475 genetically different DNA sequences was characterized to identify the functional haplotypes of *GBSSII* and a series of analyses were performed, genetic diversity and population genetic structure analyses to gain evolutionary insight into this gene, *GBSSII*, based on the classified populations.

Haplotype analysis of a specific gene reveals its distinct major dormant and non-dormant haplotypes [52] and can also identify many functional alleles which are significantly associated with desired plant traits required for a breeding program, such as eating quality or salt tolerance and resistance [53]. Genome-wide haplotype analysis of the starch synthase gene family recently conducted in rice resulting in the identification of six haplotypes with 17 SNPs were identified in the diverse regions of *GBSSII* [20]. In our study, a total of 38 functional haplotypes was recognized, representing several nonsynonymous substitutions (30 SNPs) within the diverse regions of *GBSSII* (Figure 1). Among the three groups of haplotype collections, the cultivated group had not only major haplotypes (C1 and C_3) but also had 19 nonsynonymous SNPs across the different *GBSSII* coding regions. The most predominant haplotype, C_1 had only a single amino acid change of leucine (L) to serine (S) resulting from an A-G substitution at position 12,917,914 on chromosome 7, while the C_3 haplotype had multiple alleles with non-synonymous substitutions across multiple exons in 20 temperate japonica rice accessions. In the wild group, 21 haplotypes revealed 20 SNP positions within the *GBSSII* gene region, representing a total of 30 wild rice accessions. Another major haplotype (M_3) was found in the mixed group, including a total of 93 rice accessions among which indica predominated. Similar to C_3, M_3 exhibited nonsynonymous SNPs. These findings in our study were consistent with the previous findings from a *GBSSII* haplotype analysis that found that Hap_4 and Hap_5 were the predominant haplotypes of *GBSSII* haplotyping and the respective rice accessions were mostly from two major rice groups (indica in Hap_4 and japonica in Hap_5) compared to other groups, aus and admixture [20]. Simultaneously, that research group identified a key *GBSSII* SNP (T/G), which produces significant variations in amylose content. In our study, many functional *GBSSII* alleles (19 SNPs in 13 cultivated haplotypes and 20 SNPs in 21 wild haplotypes) were identified. These functional genetic variations (SNPs) can be assumed to be of high consideration in molecular breeding of starch-related rice varieties’ improvement programs.

The levels of starch accumulation and free sugars in rice leaves (sheaths and culm) can be a maximum within 10 to 11 weeks after transplanting [54] and these sugars induce the accumulation of *OsGBSSII* transcripts through the glycolysis-dependent pathway [7]. These accumulated starches in rice leaves are then rapidly decreased during grain filling mechanism [54] and are indirectly translocated into the panicles as carbohydrate contents for grain development [55,56,57]. Starch content in mature grain can be grain-filling-duration dependent [58] and it can also be regulated by a starch remobilization mechanism in leaf sheaths after rice heading [59]. Therefore, starch biosynthesis in transient organs (leaves) directly or indirectly appeared to be correlated with structural and behavioral functionalities of starch storage (seeds). In our study, functional studies of *GBSSII* haplotypes and their responsible alleles were detected, and these findings can be assumed to be key enzymes for transitory starch. Despite its main expression in transient organs during the daytime, synthetic starches are mobilized at night for plant carbohydrates regulated by circadian rhythm, which indirectly supply non-structural carbohydrates (NSCs) for grain filling. One recent research reported that remobilization of stored starch from leaves to developing grains was positively correlated with a decrease of starch and starch synthase (SS) activities in leaves [58]. Understanding the starch mechanism and its synthetic genes expressed in different plant tissues is beneficial to comprehensive study of the starch synthase gene family in rice. Simultaneously, characterization of these genes in their genetic controls for functional activities also appears to be indispensable.

High-throughput RNA sequencing (RNA-Seq) provides insight into the transcriptome [60], and careful selection of the RNA-Seq quantification measure is critical for inter-sample comparisons, such as different gene expression levels between two or more conditions [61]. In this study, the measures of fragments per kilobase of exon per million (FPKM) were quantified from the RNA-Seq data of 353 rice accessions (300 cultivated and 53 wild) within the *GBSSII* genomic region for an expression analysis based on the identified haplotypes. Association testing using a generalized linear model (GLM) through FPKM measures generated 19 marker positions for *GBSSII* expression (Data S4), of which only three positions (one SNP and two InDels) overlapped from those from haplotyping (S5). The haplotypes representative of rice accessions identified from the FPKM measures were investigated for variations (Figure S6). The results revealed a significant difference between the M_3 haplotype (indica specific) and the C_3 haplotype (temperate japonica specific), indicating a higher level of *GBSSII* expression in temperate japonica. However, most of the observed variants (SNPs and InDels) found in these haplotypes (M_3 and C_3) were almost the same (Data S5). Despite the differences in the variant number among the other haplotypes, no significant difference was detected in their expression levels.

Many recent studies have shed-light on genetic diversity and molecular evolution in rice in terms of analyzing gene regions and improving varietal quality [62,63,64,65]. Of which, many starch synthesis genes have been studied on their genetics and genomics [47,66] but *GBSSII* is the only exception. Analysis of nucleotide diversity is important to highlight the functional importance of genomic regions [67] and it has also appeared to be useful to characterize local populations, representing the whole genome distribution of each population using π-values [68]. In our study, the nucleotide diversity analysis based on the classified ecotypes of 475 rice accessions revealed the lowest diversity values in japonica ecotypes (temperate japonica, 0.0029, and tropical japonica, 0.0030) despite the wild group representing its lowest diversity value (0.0045) on a variety basis, suggesting no significant differences between the wild group and any of the identified cultivated subgroups (Figure 3 and Table S3). However, the relative lowest diversity values of japonicas were assumed to be domestication signatures under selective sweeps due to genetic bottleneck effects. Previous findings suggested that lower genetic diversity was experienced in a recent bottleneck during the domestication selection of the waxy locus in the waxy rice genome [69,70,71]. On the other hand, the remaining cultivated subgroups, indica, aus, and admixture (representing higher diversity landrace, weedy, and bred groups in another way) were higher in nucleotide diversity than that of the wild group, suggesting positive artificial selection due to the high π-value regions in each of these classified populations [68].

Tajima’s *D*-values together with nucleotide diversity and linkage disequilibrium (LD), are the estimators of the domestication signatures of the crops compared to their wild progenitors [72,73,74,75]. Referring to Tajima’s *D* values, the molecular evolution of the starch synthetic genes, such as *AGPL2*, *AGPS2b*, *SSIIa*, *SBEIIb*, *GBSSI*, and *ISA1*, has been estimated in rice endosperm under a standard neutral model [70,76,77,78,79]. The Tajima’s *D* results in our study revealed a negative value for the wild group compared to the other cultivated variety groups of landrace, weedy, and bred. In the case of ecotype-based analysis, the wild group, as well as both japonicas (temperate and tropical), revealed negative Tajima’s *D* estimates, compared to the other classified ecotypes of indica, aus, and the admixture. These negative *D* values in the japonica and wild groups occurred because of an excess of rare alleles within this gene region compared to expectations, indicating evidence for purifying selection. This consistent feature of selective force has been shown in a molecular evolution analysis of the *ALK* (*SSIIa*) gene using 321 rice accessions [77]. However, the landrace, weedy, and bred varieties (in terms of ecotypes by indica, admixture, and aus) expressed positive Tajima’s *D* values within the *GBSSII* region, subjected to balancing selection through signifying higher observed diversity compared to those of the expected values.

Population structure is a systematic pattern used to measure the relatedness between individuals in large genomic data sets together with the dimension reduction technique such as principal component analysis (PCA) to visualize the principal components [80]. The structure clustering analysis demonstrated a clear separation of the classified populations (varieties or ecotypes, including the wild group) at most of K values (3–7). However, all of their internal subgroups were admixed, suggesting a diverse relationship among them due to their probable differing ancestral sources. PCA and the phylogenetic analysis also indicated similar features of the relationships among the classified populations where cultivated populations were interbred. Despite the clear separation of the four major groups by PCA, each group was composed of many classified populations. However, in variety-based PCA, group II showed a close association between the wild and bred groups which appeared to be in recent ancestors in the phylogenetic analysis by means of temperate japonica subclade between the two wild subclades. Forty-five haplotypes formed a network that infers their relatedness and estimates the recent evolutionary history of these diverse populations within the *GBSSII* region. Like the wild haplotypes, the cultivated haplotypes also expressed a diverse pattern, indicating close and similar genetics among their internal subgroups. Despite the vector-mediated connection between the wild and cultivated haplotypes, they were inferred to have a recent evolutionary history due to their associations with fewer mutational steps.

The empirical Bayesian inference of pairwise *F_ST_* and its distribution in the genome has been used to estimate the locus-specific pairwise *F_ST_* [81], and it is a widely used descriptive statistic in evolutionary genetics to provide insights based on genetic variations within and among populations [82]. In our study, both the highest and lowest genetic differentiation values of the *GBSSII* locus were observed between the cultivated ecotypes (0.8313 for the tropical japonica-aromatic pair and 0.0498 for temperate-tropical pair). However, the bred group was markedly distant from the wild group based on an *F_ST_*-value of 0.1992, compared to the other varieties. A population structure and diversity analysis of red rice germplasm indicated a similar genetic differentiation feature among the populations, with pairwise *F_ST_* values of 0.108–0.207 [83]. Our findings also indicate a variety of greater differentiations among most of the pairs (60%) of classified ecotypes.

Identifying genes of interest is vital for conducting a successful breeding program and improving agronomic traits [84]. Functional alleles are very helpful to such breeding programs, for example, a functional SNP (G/A in exon 3) of the *SSIIIa* gene is useful for eating and cooking quality performance [84] and two waxy SNPs (G/T in intron 1 and A/C in exon 6) are useful for selecting the desired amylose content in rice varieties [13]. The haplotype analysis in our study provided many functional *GBSSII* alleles potentially suitable for future breeding studies by estimating the diverse structures of *GBSSII* and providing evolutionary insight among or between the classified rice groups.

## 5. Conclusions

Our findings provide collective and supportive information for the functional haplotypes of *GBSSII* in cultivated (13 haplotypes with 19 nsSNPs) and wild (21 haplotypes with 20 nsSNPs) rice accessions. These functional haplotypes identifying their specific alleles together with their detected regions can be of applicable and valuable genetic information for further studies of this gene performance in amylose biosynthesis (transient starch). Estimations of domestication signatures through a series of evolutionary analyses (genetic diversity, population genetics structure, and evolutionary relationships in phylogeny and differentiation analyses) provide different selective sweeps (balancing selection and purifying selection), which appear to be useful in molecular breeding programs for this gene’s functions. These findings of our study can promote further research in a variety development with desired traits activated by this gene.

## Figures and Tables

**Table 1:** Summary of genetic variations in the *GBSSII* gene region of 475 accessions from the Korean world rice collection. The *GBSSII* reference gene region was adapted from Nipponbare. Others ^(1)^: group of wild rice accessions other than *Oryza nivara* and *Oryza rufipogon*, Ins ^(2)^: insertion, Del ^(3)^: deletion, Dupl ^(4)^: duplication, and DV ^(5)^: different variation.

Group	Subgroup (Ecotype)	Total No. of Variations					No. of Accessions
		SNP	Ins ^(^^2)^	Del ^(^^3)^	Dupl ^(^^4)^	DV ^(^^5)^	
Cultivated rice	Temperate Japonica	193	16	21	1	1	279
	Tropical Japonica	122	3	7	0	0	26
	Indica	196	13	15	1	1	102
	Aus	200	12	16	1	1	9
	Aromatic	101	4	5	0	0	2
	Admixture	146	6	10	0	1	3
Wild rice	*O. nivara*	121	5	5	0	0	3
	*O. rufipogon*	120	6	9	1	0	3
	Others ^(1)^	276	25	43	1	0	48

## Data Availability

The data is available within the article or Supplementary Materials.

## Supplementary Materials

The following are available online at https://www.mdpi.com/article/10.3390/foods10102359/s1, Data S1: Passport information of the 475 Korean rice accessions (KRICE_CORE) used in this study, Data S2: List of the 475 Korean rice accessions indicating raw data for all genetic variations (SNPs and InDels) within the *GBSSII* gene region by haplotyping. Haplotype analysis generated a total of 59 haplotypes representing a total of 425 positions by 370 SNPs (single nucleotide polymorphism) and 55 InDels (insertions or deletions) detected in both exons and introns including UTR (untranslated regions) of 5’ and 3’. Rows for codon and amino acid (AA) were supported for the positions where nonsynonymous SNP (SNP substitutions) in exons were identified, Data S3: Haplotyping revealed a list of 45 haplotypes representing a total of all identified 113 variants, including 58 SNPs (single nucleotide polymorphism) and 55 InDels (insertions or deletions) within the gene region of *GBSSII* in the 475 Korean rice accessions. Detected variants were summarized from both exons and introns including UTR (untranslated regions) of 5′ and 3′. Rows for codon changes together with amino acid (AA) substitutions were supported for the positions where nonsynonymous SNP (SNP substitutions) were located, Data S4: List of positions identified by a generalized linear model (GLM) using fragments per kilobase of exon million (FPKM) measures of transcript through RNA-Seq (RNA sequencing) of 353 Korean rice collections (300 cultivated and 53 wild) within the *GBSSII* gene region. Only the highlighted (light-blue color) positions (19 positions showing higher *p*-values) were selected as marker positions to be analyzed for the *GBSSII* gene expression. These 19 positions were investigated for their significant response to gene expression based on the identified predominant haplotypes, Data S5: List of haplotypes representing identified SNP and InDel positions not only from haplotype analysis but also from significant associated positions for RNA expression in generalized linear model within the *GBSSII* gene region. The analysis was based only on identified positions. Yellow-colored positions were those identified from RNA expression data. Cyan (for SNPs) and orange-colored (InDels) positions were those identified from haplotype grouping. Blue-colored positions were those overlapped among the positions of both expression and haplotype analyses. Highlighted alleles were minor alleles identified in their respective positions, Table S1: Summary of the 475 Korean rice accessions based on their different varietal types or ecotypes, Table S2: Pairwise estimates of genetic differentiation (*F_ST_* values) of the *GBSSII* gene between different subgroups of 475 Korean rice collections. Te_Japonica ^(1)^: temperate japonica, Tr_Japonica ^(2)^: tropical japonica, Table S3: Summary of average nucleotide diversity (π) and Tajima’s *D* values within the *GBSSII* gene region of the 475 Korean rice accessions by means of different variety types, Table S4: Summary of average nucleotide diversity (π) and Tajima’s *D* values within the *GBSSII* gene region of the 475 Korean rice accessions by means of different ecotypes, Figure S1: Estimates of structure and population differentiation within the gene region of *GBSSII* (*Os07g0412100*) in the 475 Korean rice accessions based on ecotypes (temperate japonica, indica, tropical japonica, aus, aromatic and admixture) including the wild. (A) Population structure of the *GBSSII* gene in the 475 Korean rice accessions clustered by increasing K values from 2 to 7. Different colors of each K value refer to different numbers of clustered populations. (B) Two-dimensional (2D) principal component analysis (PCA) of the 475 Korean rice accessions. (C) Pairwise estimates of genetic differentiation (*F_ST_* values) of the *GBSSII* gene among the different ecotypes of the 475 Korean rice accessions, Figure S2: Nucleotide diversity analysis of the *GBSSII* (*Os07g0412100*) gene in the 475 Korean rice accessions by means of ecotypes (temperate japonica, indica, tropical japonica, aus, aromatic and admixture) together with the wild group. (A) Nucleotide diversity (π-value) representing the number of nucleotide variations within the *GBSSII* gene region at individual segregating sites in 1.5 kb sliding window. Cyan indicates the *GBSSII* gene region, and each colored line represents an individual ecotype. (B) Box plots representing the different distribution patterns of *GBSSII* genetic variations based on mean nucleotide diversity values among the classified ecotypes, Figure S3: Tajima’s *D* values of *GBSSII* (*Os07g0412100*) in the 475 Korean rice accessions by means of ecotypes (temperate japonica, indica, tropical japonica, aus, aromatic and admixture) together with the wild. (A) Tajima’s *D* values representing different individual segregating sites within the *GBSSII* gene region in 1.5 kb sliding window. Cyan indicates the *GBSSII* gene region, and different colored lines represent different rice ecotypes. (B) Box plots represent different distribution patterns of *GBSSII* genetic variations according to Tajima’s *D* values among the ecotypes, Figure S4: A list of 38 haplotypes representing 55 InDel variations within *GBSSII* region of 475 rice accessions. Most of the variations were deletions (Dels) and very few positions were identified for insertions (Ins), Figure S5: Phylogenetic tree for the orthologous genes *GBSS1* and *GBSSII*. Scale bar indicates the proportion of sites changing along each branch. To characterize the relationship between *GBSS1* and *GBSSII*, these homologues in rice and other plant species were inferred using ORTHOFINDER [85]. Nineteen different plant species (most were from rice) were used from these public databases, https://rice-genome-hub.southgreen.fr/node/70/53; Ensembl Plants; http://rice.hzau.edu.cn; and https://rapdb.dna.affrc.go.jp, last accessed on 20 April 2021, Figure S6: Statistical analysis on the association between significant marker positions by a generalized linear model (GLM) using fragments per kilobase of exon per million (FPKM) measures and those detected under selected haplotypes by haplotype analysis. The hypothesis was performed by Scheffé test at alpha value by 0.05 (5%) and the selection of haplotypes were based on the rice accession numbers (≥5) each haplotype belonged to.
