# Supplementary material for: Functional Haplotypes and Evolutionary Insight into the Granule-Bound Starch Synthase II (GBSSII) Gene in Korean Rice Accessions (KRICE_CORE)
Source: Foods. 2021 Oct 3;10(10):2359. doi: 10.3390/foods10102359 (PMC8535093; doi:10.3390/foods10102359)
Supplement: Supplementary file 1 [file foods-10-02359-s001.zip › Supplementary Materials_R1.pdf]

Supplementary Materials

Supplementary Data

Data S1: Passport information of the 475 Korean rice accessions (KRICE_CORE) used in this study

Data S2: List of the 475 Korean rice accessions indicating raw data for all genetic variation (SNPs and InDels) within the *GBSSII* gene region by haplotyping. Haplotype analysis generated a total of 59 haplotypes representing a total of 425 positions by 370 SNPs (single nucleotide polymorphism) and 55 InDels (Insertions or Deletions) detected in both exons and introns including UTR (untranslated regions) of 5' and 3'. Rows for codon and amino acid (AA) were supported for the positions where nonsynonymous SNP (SNP substitutions) in exons were identified

Data S3: Haplotyping revealed a list of 45 haplotypes representing a total of all identified 113 variants, including 58 SNPs (single nucleotide polymorphism) and 55 InDels (Insertions or Deletions) within the gene region of *GBSSII* in the 475 rice accessions. Detected variants were summarized from both exons and introns including UTR (untranslated regions) of 5' and 3'. Rows for codon changes together with amino acid (AA) substitutions were supported for the positions where nonsynonymous SNP (SNP substitutions) were located

Data S4: List of positions identified by a generalized linear model (GLM) using fragments per kilobase of exon million (FPKM) measures of transcript through RNA-Seq (RNA Sequencing) of 353 Korean rice collection (300 cultivated and 53 wild) within the *GBSSII* gene region. Only the highlighted (light-blue color) positions (19 positions showing higher *p*-values) were selected as marker positions to be analyzed for the *GBSSII* gene expression. These 19 positions were investigated for their significant response to gene expression based on the identified predominant haplotypes

Data S5: List of haplotypes representing identified SNP and InDel positions not only from haplotype analysis but also from significant associated positions for RNA expression in generalized linear model within the *GBSSII* gene region. The analysis was based on only identified positions. Yellow-colored positions were those identified from RNA expression data. Cyan (for SNPs) and Orange-colored (InDels) positions were those identified from haplotype grouping. Blue-colored positions were those overlapped among the positions of both expression and haplotype analyses. Highlighted alleles were minor alleles identified in their respective positions

Supplementary Tables

Table S1: Summary of the 475 Korean rice accessions based on their different varietal types or ecotypes

Varietal Type	No. of Rice Accessions Occupied		Ecotype
Landrace	49	45	Temperate Japonica
		20	Indica
		9	Tropical Japonica
Weedy	28	2	Aus
		1	Admixture
Bred	320	227	Temperate Japonica
		70	Indica
		14	Tropical Japonica
		5	Aus
		2	Aromatic
		2	Admixture
Wild	54		
Unknown	24	7	Temperate Japonica
		12	Indica
		3	Tropical Japonica
		2	Aus
Total	475	475	

Table S2: Pairwise estimates of genetic differentiation (F_{ST} values) of the *GBSSII* gene between different subgroups of 475 Korean rice collection. Te_Japonica[1)]: temperate japonica, Tr_Japonica[2)]: tropical japonica

Subgroups	Te_Japonica	Tr_Japonica	Indica	Aus	Aromatic	Admixture	Wild
Te_Japonica[1)]	-						
Tr_Japonica[2)]	0.0498	-					
Indica	0.6952	0.6935	-				
Aus	0.5678	0.5560	0.3122	-			
Aromatic	0.7567	0.8318	0.2412	0.1029	-		
Admixture	0.1367	0.2504	0.6263	0.1176	0.6548	-	
Wild	0.4333	0.3748	0.4335	0.1343	0.2468	0.1677	-

Table S3: Summary of average nucleotide diversity (π) and Tajima's *D* values within the *GBSSII* gene region of the 475 Korean rice accessions by means of different varietal types

Varietal Type	Parameter	Value
Landrace	Nucleotide Diversity (π value)	0.0036
	Tajima's D value	0.2764
Weedy	Nucleotide Diversity (π value)	0.0033
	Tajima's D value	1.5286
Wild	Nucleotide Diversity (π value)	0.0056
	Tajima's D value	-0.0403
Bred	Nucleotide Diversity (π value)	0.0013
	Tajima's D value	-1.0488

Table S4: Summary of average nucleotide diversity (π) and Tajima's D values within the *GBSSII* gene region of the 475 Korean rice accessions by means of different ecotypes

Ecotypes	Parameter	Value
Temperate Japonica	Nucleotide Diversity (π value)	0.0003
	Tajima's D value	-0.3401
Tropical Japonica	Nucleotide Diversity (π value)	0.0010
	Tajima's D value	-1.0801
Indica	Nucleotide Diversity (π value)	0.0044
	Tajima's D value	1.8349
Aus	Nucleotide Diversity (π value)	0.0016
	Tajima's D value	0.4665
Admixture	Nucleotide Diversity (π value)	0.0049
	Tajima's D value	0.9264
Wild	Nucleotide Diversity (π value)	0.0056
	Tajima's D value	-0.0403

Supplementary Figures

Figure S1: Estimates of structure and population differentiation within the gene region of *GBSSII* (*Os07g0412100*) in the 475 Korean rice accessions based on ecotypes (temperate japonica, indica, tropical japonica, aus, aromatic and admixture) including the wild. **(A)** Population structure of the *GBSSII* gene in the 475 Korean rice accessions clustered by increasing K values from 2 to 7. Different colors of each K value refer to different numbers of clustered populations. (**B)** Two-dimensional (2D) principal component analysis (PCA) of the 475 Korean rice accessions. **(C)** Pairwise estimates of genetic differentiation (F_{ST} values) of the *GBSSII* gene among the different ecotypes of the 475 Korean rice accessions

Figure S2: Nucleotide diversity analysis of the *GBSSII* (*Os07g0412100*) gene in the 475 Korean rice accessions by means of ecotypes (temperate japonica, indica, tropical japonica, aus, aromatic and admixture) together with the wild group. **(A)** Nucleotide diversity (π-value) representing the number of nucleotide variations within the *GBSSII* gene region at individual segregating sites in 1.5 kb sliding window. Cyan indicates the *GBSSII* gene region, and each colored line represents to an individual ecotype. **(B)** Box plots representing the different distribution patterns of *GBSSII* genetic variations based on mean nucleotide diversity values among the classified ecotypes

Figure S3: Tajima's *D* values of *GBSSII* (*Os07g0412100*) in the 475 Korean rice accessions by means of ecotypes (temperate japonica, indica, tropical japonica, aus, aromatic and admixture) together with the wild. **(A)** Tajima's *D* values representing different individual segregating sites within the *GBSSII* gene region in 1.5 kb sliding window. Cyan indicates the *GBSSII* gene region, and different colored lines represent different rice ecotypes. **(B)** Box plots represent different distribution patterns of *GBSSII* genetic variations according to Tajima's D values among the ecotypes

Figure S4: A list of 38 haplotypes representing 55 InDel variations within *GBSSII* region of 475 rice accessions. Most of the variations were deletions (Dels) and very few positions were identified for insertions (Ins)

Figure S5: Phylogenetic tree for the orthologous genes *GBSS1* and *GBSSII*. Scale bar indicates the proportion of sites changing along each branch. To characterize the relationship between *GBSS1* and *GBSSII*, these homologues in rice and other plant species were inferred using ORTHOFINDER [80]. Nineteen different plant species (most were from rice) were used from these public databases, https://rice-genome-hub.southgreen.fr/node/70/53; https://rapdb.dna.affrc.go.jp Ensembl Plants and http://rice.hzau.edu.cn, last accessed on 15 June 2021

Figure S6: Statistical analysis on the association between significant marker positions by a generalized linear model (GLM) using fragments per kilobase of exon per million (FPKM) measures and those detected under selected haplotypes by haplotype analysis. The hypothesis was performed by Scheffé test at alpha value by 0.05 (5%) and the selection of haplotypes were based on the rice accession numbers (≥ 5) each haplotype belonged to.
