# Supplementary material for: Functional Haplotypes and Evolutionary Insight into the Granule-Bound Starch Synthase II (GBSSII) Gene in Korean Rice Accessions (KRICE_CORE)
Source: Foods. 2021 Oct 3;10(10):2359. doi: 10.3390/foods10102359 (PMC8535093; doi:10.3390/foods10102359)
Supplement: Supplementary file 1 [file foods-10-02359-s001.zip › S_Figure_S4.pdf]

Different Groups of Haplotypes	Chromosome	12,917,071	12,917,159	12,917,336	12,917,339	12,917,410	12,919,733	12,919,857	12,919,862	12,919,915	12,920,751	12,921,224	12,922,265	12,922,396	12,922,614	12,922,870	12,922,937	12,923,103	12,923,160	12,923,218	12,923,497	12,923,584	12,923,643	12,923,707	12,923,727	12,923,876	12,923,983	12,924,022
	7	G	G	C	G	G	G	A	G	C	A	C	G	T	A	A	A	A	A	A	C	A	A	C	T	A	T	A
	OS07G0412100	<	<	<	<	<	<	<	<	<	<	<	<	<	<	<	<	<	<	<	<	<	<	<	<	<	<	<
	Reference / Haplotype	GATACAAAT	GGCACC	CTA	GCT	GTATATATTACAAATTGAACAAATAACTTAAAAT	GATAA	ATTCT	GTTCTC	CAAA	ATCTG	CAGTGTGCTCATCTAATCCAA	GGC	TTA	AGG	AGG	ATG	ACCC	AATAT	ATTT	CCTT	AGTTAAT	AAAATAT	CAGTACT	TAA	ACATACTC	TCC	ACTGGTGCAG
Mixed	M_1	0	0	0	0	0	0	0	0	0	0	0		0 -2	0 -2	0	0	0 -3	0	0	0	0	0	0	0	0	-2	0
	M_2	0	0	0	0	0	0	0	0	0	0	0	0 -2	0 -2	-2	0	0	0	0	-3	0	0	-6	0	0	0	-2	0
	M_3	0	0	0	0	-33	0	0	0	0	0	0		0 -2	-2	0	0	3	0	-3	0	0	-6	0	0	0	-2	0
	M_4	0	0	0	0	-33	0	0	0	0	0	0	0 -2	0 -2	-2	0	0		0	-3	0	0	-6	0	0	0	-2	0
Cultivated Rice	C_1	0	0	0	0	0	0	0	0	0	0	0	0	0	0	0	0	0	0	0	0	0	0	0	0	0	-1	0
	C_2	0	0	0	0	0	0	0	0	0	0	0	0 -2	0 -2	0 -2	0 -2	0 -2		0	0	0	0	0	0	0	0	-2	0
	C_3	0	0	0	0	0	0	0	0	0	0	0	0	0 -2	-2	0	0		0	-3	0	0	-6	0	0	0	-2	0
	C_4	0	0	0	0	0	0	0	0	0	0	0	0	0	-2	0	0		0		0	0	-6	0	0	0	-2	0
	C_5	0	0	0	0	-33	0	0	0	0	0	0	0 -2	0 -2	-2	0 -2	0		0	-3	0	0	-6	0	0	0	-2	0
	C_6	0	0	0	0	0	0	0	0	0	0	0	0 -2	0 -2	0 -2	0	0		0	0	0	0	0	0	0	0	-2	0
	C_7	0	0	0	0	0	0	0	0	0	0	0	0	0	0	0	0	0	0	0	0	0	0	0	0	0	-1	0
	C_8	0	0	0	0	0	0	0	0	0	0	0	0	0	0	0	0	0	0	0	0	0	0	0	0	0	0	0
	C_9	0	0	0	0	0	0	0	0	0	0	0	0	0	-2	0	0		0	-3	0	0	-6	0	0	0	-2	0
	C_10	0	0	0	0	0	0	0	0	0	0	0	0	0	-2	0	0		0	-3	0	0	-6	0	0	0	-2	0
	C_11	0	0	0	0	0 -33	0	0	0	0	0	0	0	0	0 -2	0	0		0		0	0	0 -6	0	0	0	0	0
	C_12	0	0	0	0	0	0	0	0	0	0	0	0 -2	0 -2	0 -2	0	0	0	0	0	0	0	0	0	0	0	-2	0
	C_13	0	0	0	0	-33	0	0	0	0	0	0	0 -2	0 -2	-2	0	0		0	-3	0	0	-6	0	0	0	-2	0
Wild Rice	W_1	0	0	0	0	0	0	0	0	0	0	0				0	0	0	0		0	0	0	0	0	0		0
	W_2	0	0	0	0	0	0	-4	-5	0	0	0	0	0	0	0	0	0 -1	-4	1	0	0	0	0	0	0	-2	0
	W_3	0	0	-2	-2	0		0		-3	0	0			0	0	0	0		0		0	0		0	0		
	W_4	0	0					0	0		0	0				0	0	0		0		0	0		0	0		
	W_5	-8								0	-4					0	0											
	W_6	0	0			0		0	0		0	0				0	0	0		0		0	0		0	0		0
	W_7	0	0	-2	-2	0		0	0		0	0				0	0			0		0	0		0	0		0
	W_8	0	0	0	0	0	0	0	0	0	0	0	0	0	0	0	0	0	0	0	0	0	0	0	0	0	-2	0
	W_9	0	0		0	0		0	0		0	0				0	0	0		0		0	0	-6	0	0		
	W_10	0				0		0	0		0	0				0	0	0				0	0		0			
	W_11	0	0			0		0	0		0					0	0			0		0	0		0	0		
	W_12	0					0					0				0	0						0			0		
	W_13	0	0	0	0	0	0	0	0	0	0	0	0	0	-2	0	0		0	-3	0	0	-6	0	0	0	-2	0
	W_14	0	0	0	0	0	0	0	0	0	0	0	0	0	-2	0	0	6		-3	0	0	-6	0	0	0	-2	0
	W_15	0	0	0	0	0	0	0	0	0	0	-20	0	0 -2	0 -2	0	0	0	0	0	-3	-6	0	0	0	-7	-2	0
	W_16	0	-5	0	0	0	0	0	0	0	0	-20		0 -2	-2	0	0		0	0	0	-6	0	0	-2	-7	-2	0
	W_17	0	0			0	-4	0			0	0				0	0	0				0	0		0	0		
	W_18	0	0			0	-4	0			0	0				0	0					0	0		0	0		-10
	W_19	0				0		0			0	0				0	0					0	0	-6	0	0		
	W_20	0	0	0	0	-33	0	0	0	0	0	0	0	0 -2	-2	0	0	0 2		-3	0	0	-6	0	0	0	-2	0
	W_21	0	0	0	0	0	0	0	0	0	0	0	0	0	0	0	0	0 -3	0	0	0	0	0	0	0	0	-2 -1	0
