# Supplementary figures and images for: Functional Haplotypes and Evolutionary Insight into the Granule-Bound Starch Synthase II (GBSSII) Gene in Korean Rice Accessions (KRICE_CORE)
Source: Foods. 2021 Oct 3;10(10):2359. doi: 10.3390/foods10102359 (PMC8535093; doi:10.3390/foods10102359)

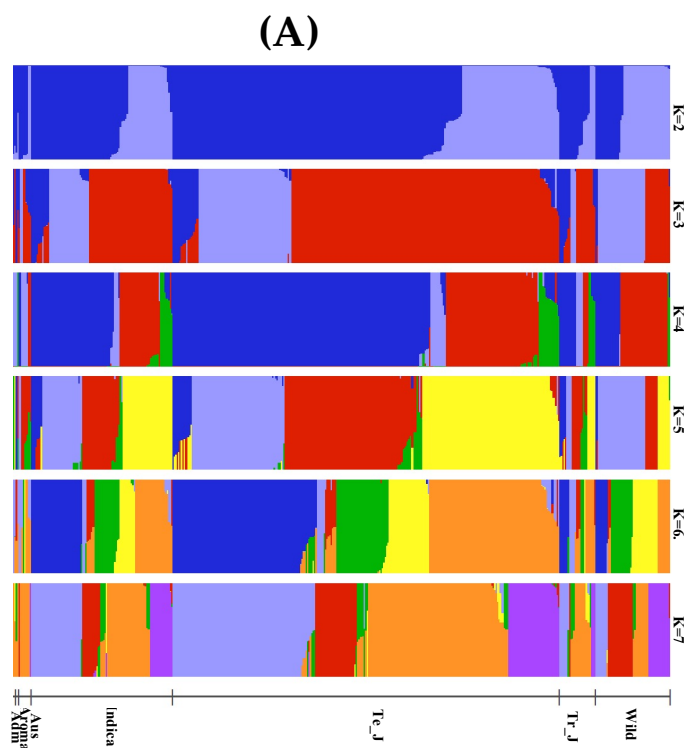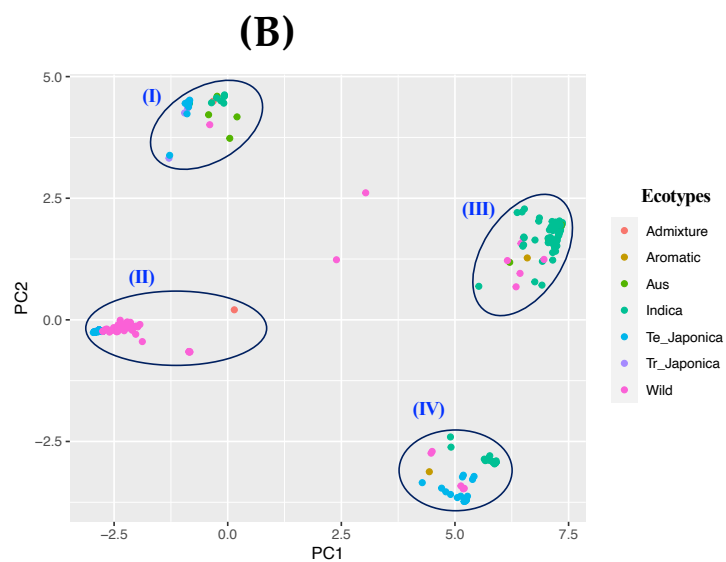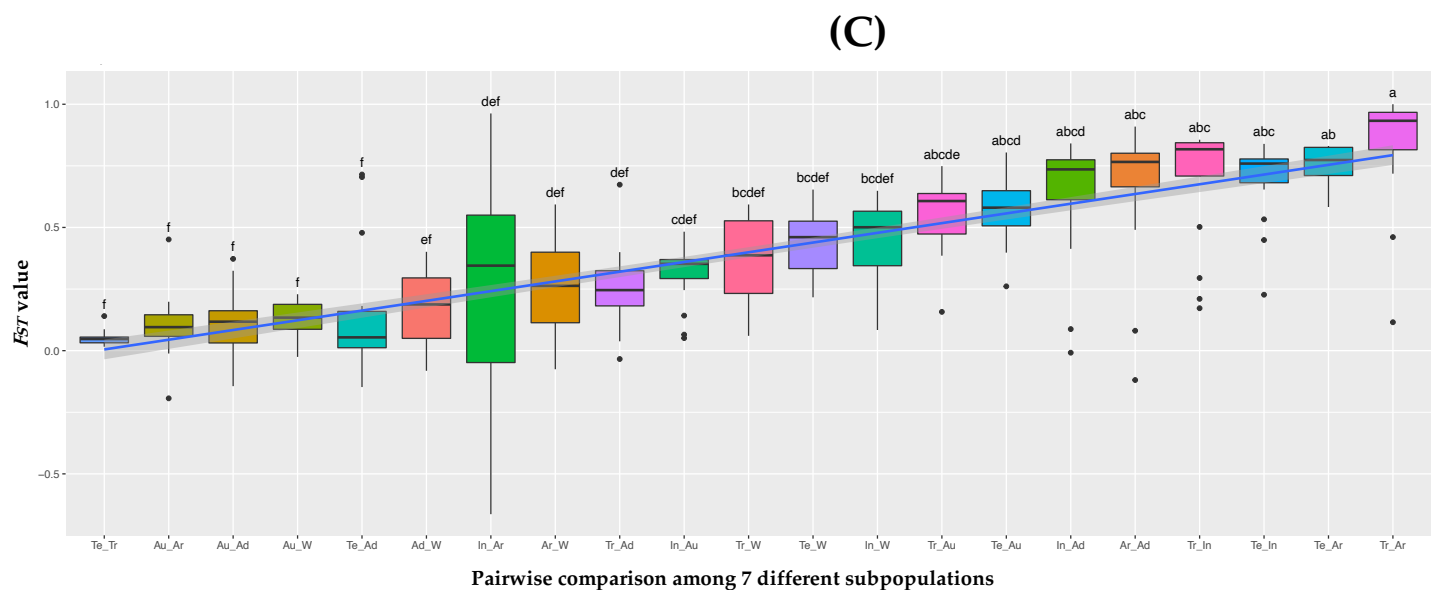

Supplement: Supplementary file 1 [file foods-10-02359-s001.zip › S_Figure_S1.pdf]

(A)

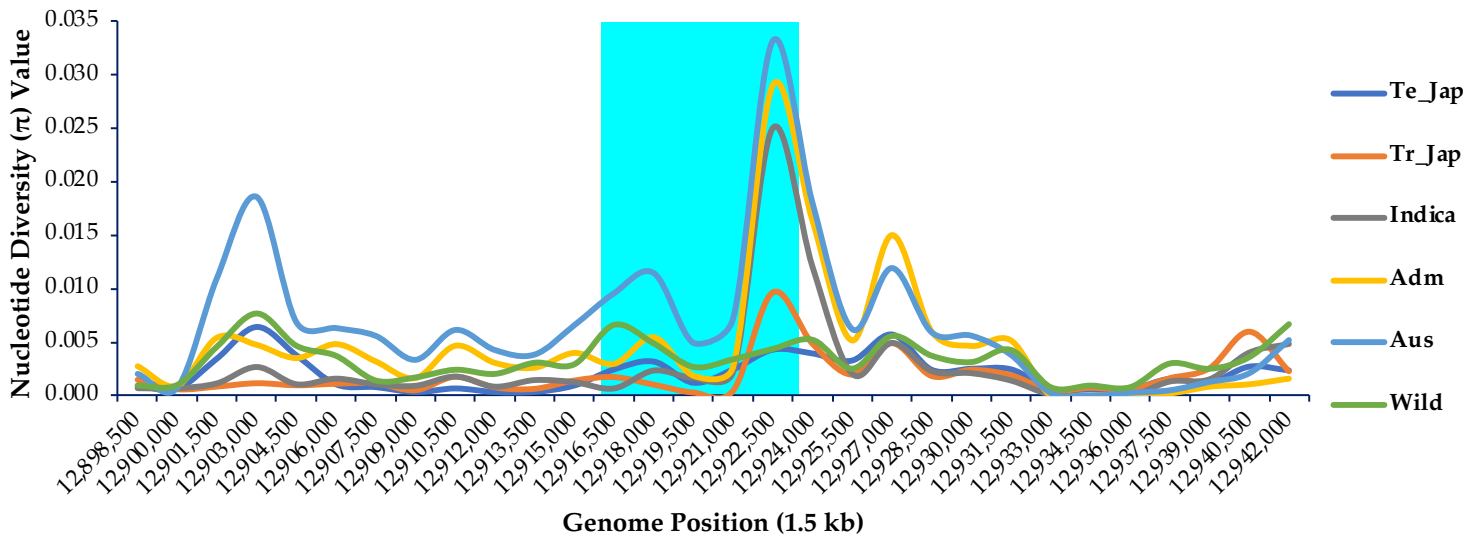

(B)

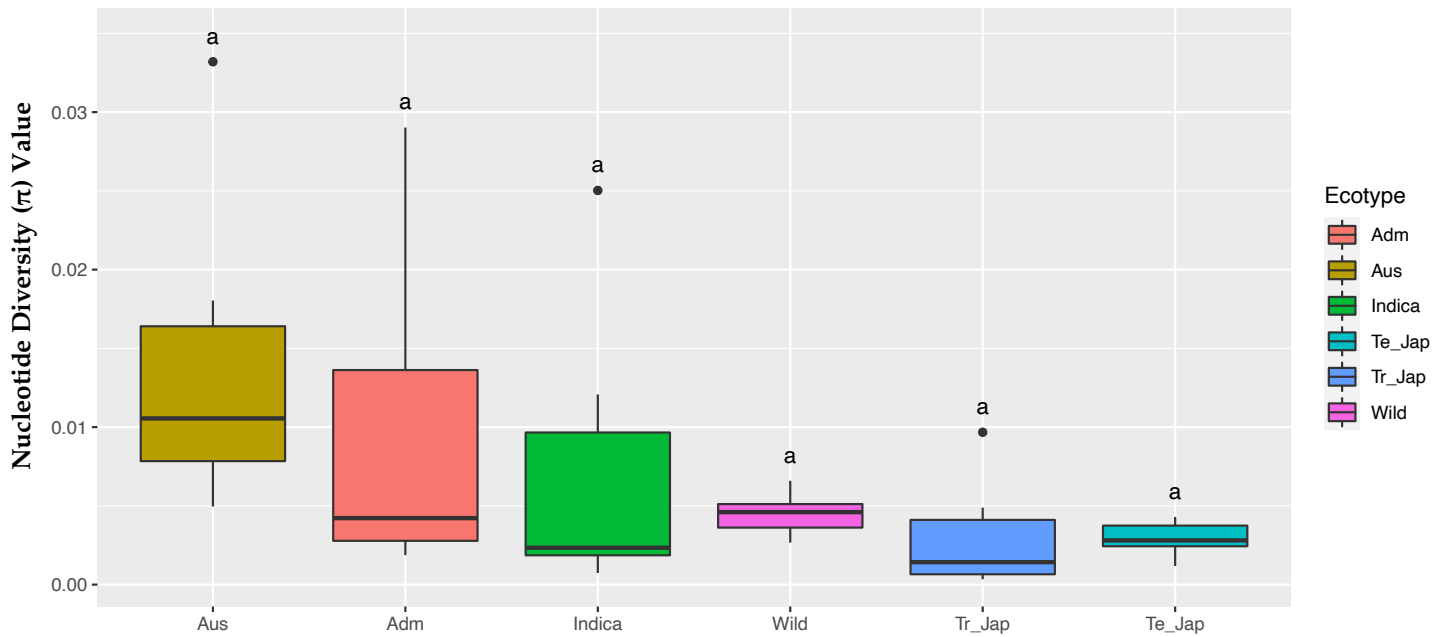

Supplement: Supplementary file 1 [file foods-10-02359-s001.zip › S_Figure_S2.pdf]

(A)

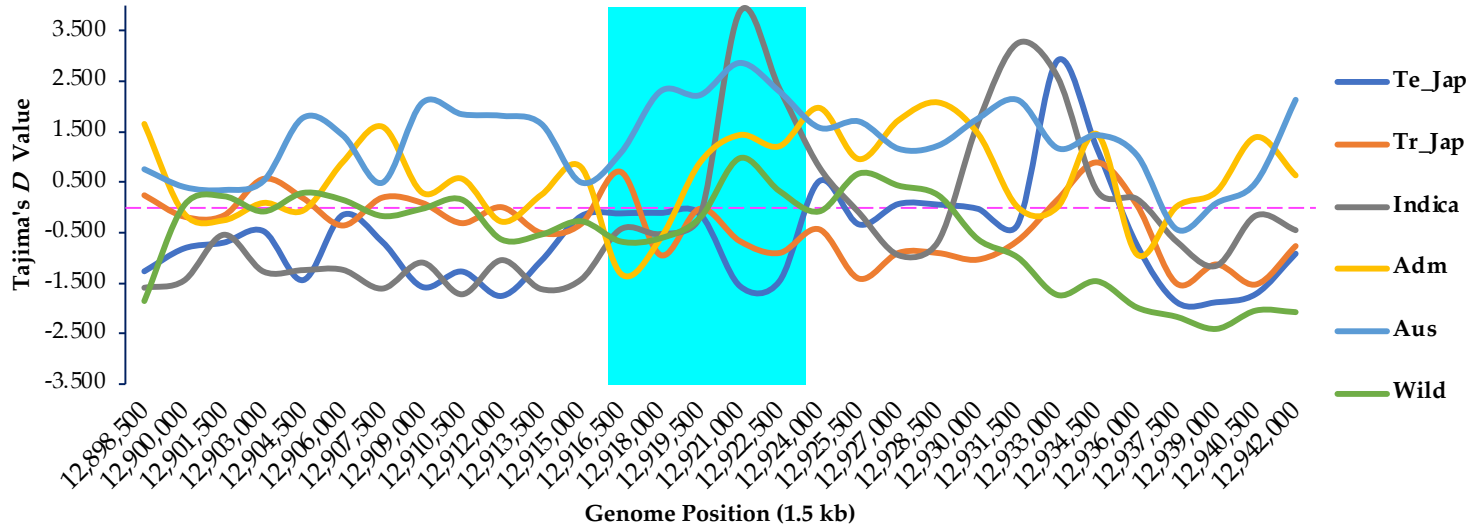

(B)

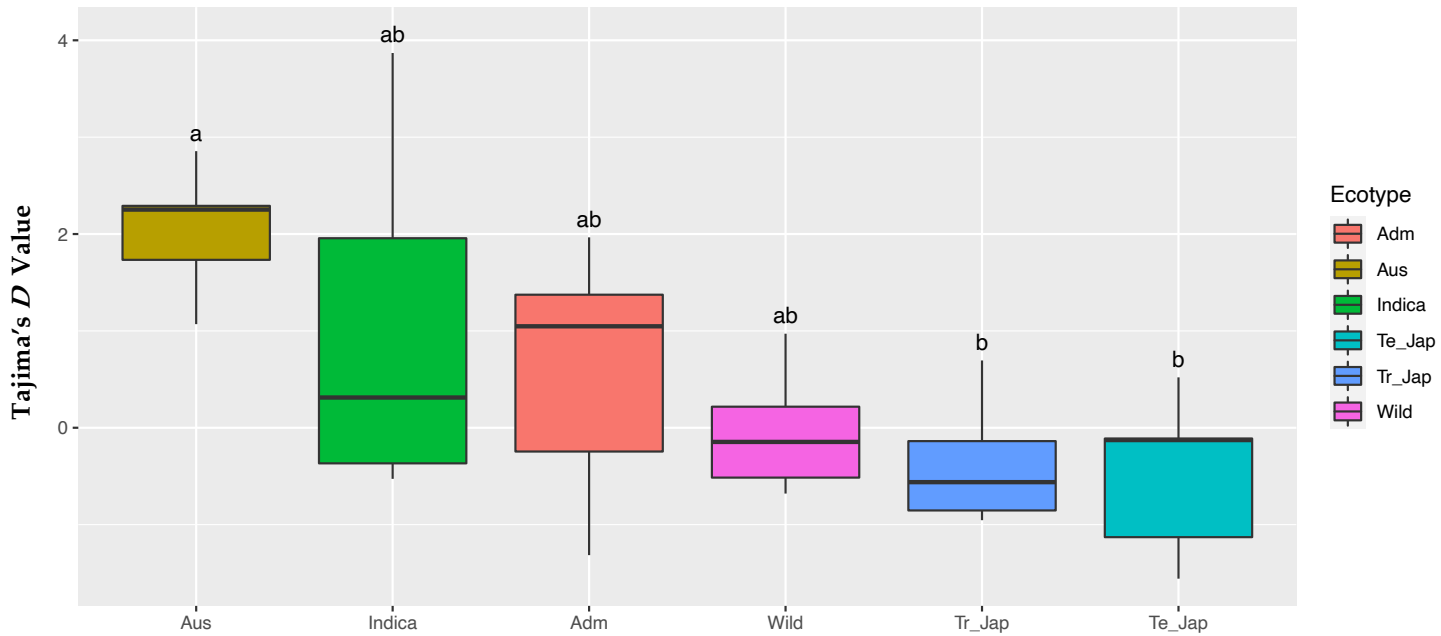

Supplement: Supplementary file 1 [file foods-10-02359-s001.zip › S_Figure_S3.pdf]

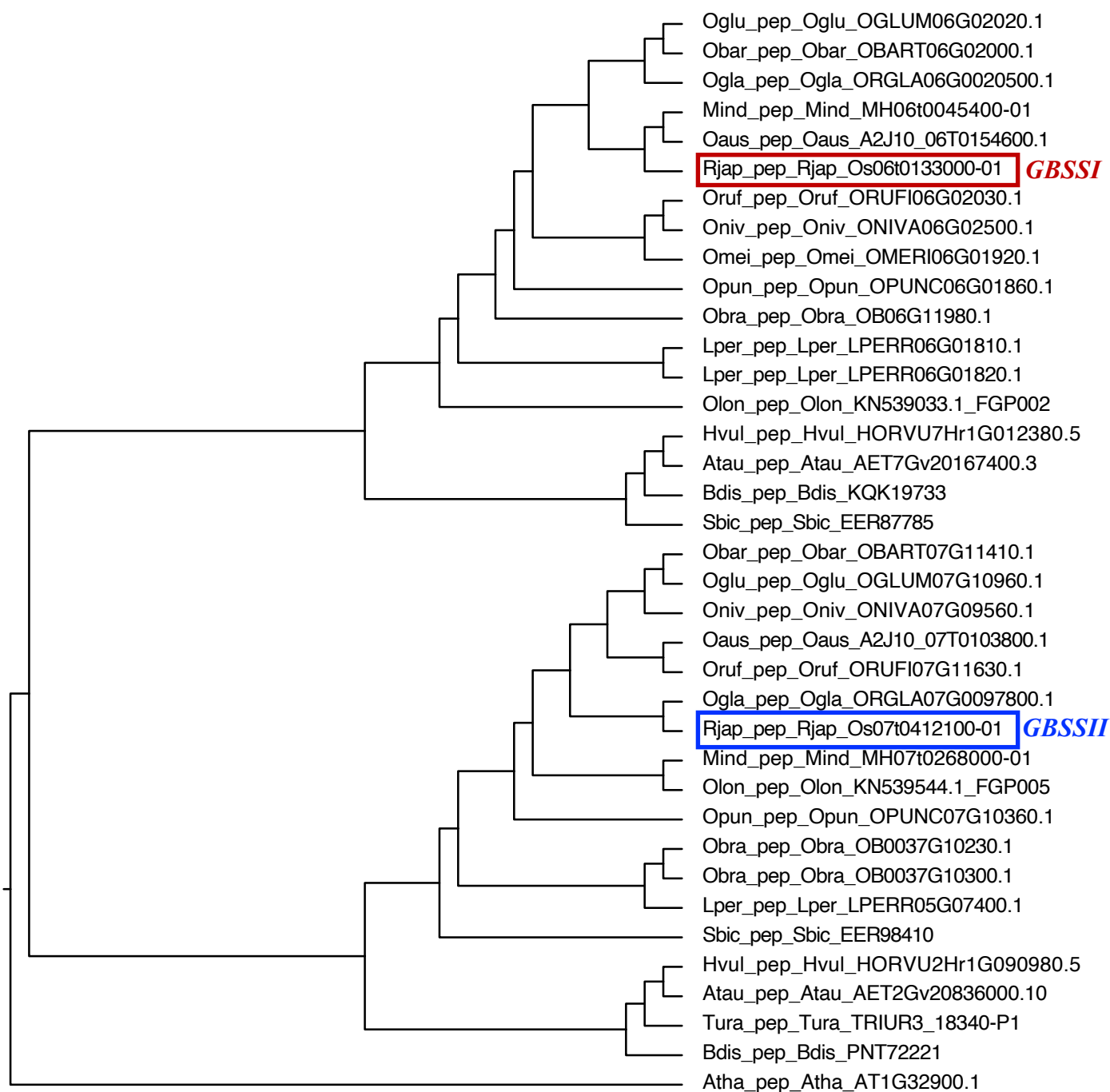

4.0

Supplement: Supplementary file 1 [file foods-10-02359-s001.zip › S_Figure_S5.pdf]

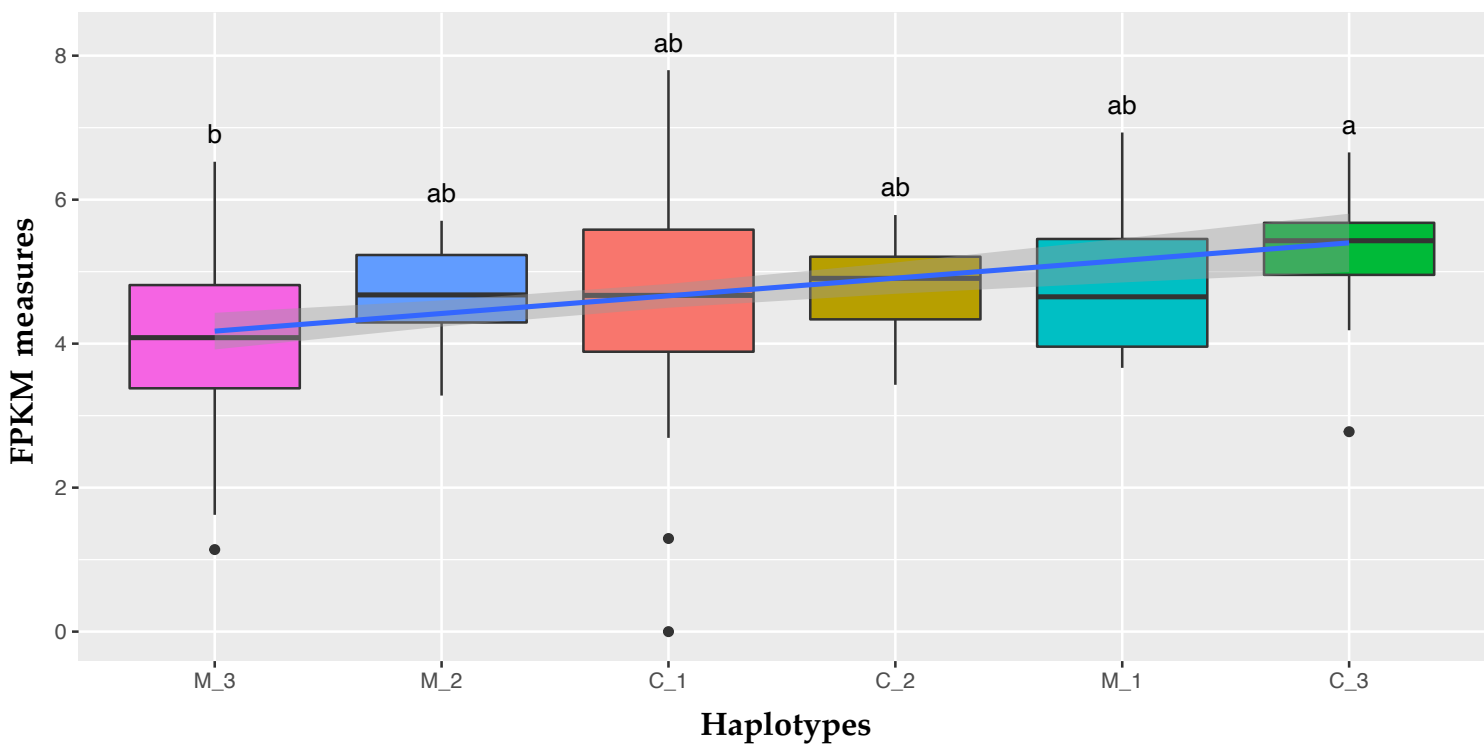

Supplement: Supplementary file 1 [file foods-10-02359-s001.zip › S_Figure_S6.pdf]
